# Quality of Life in Patients with Minimal Change Nephrotic Syndrome

**DOI:** 10.1155/2013/124315

**Published:** 2013-02-28

**Authors:** Yoshiko Shutto, Hideaki Yamabe, Michiko Shimada, Takeshi Fujita, Norio Nakamura

**Affiliations:** ^1^Hirosaki University Graduate School of Health Sciences, 66-1 Hon-cho, Aomori, Hirosaki-shi 036 8564, Japan; ^2^Department of Cardiology, Respiratory Medicine and Nephrology, Hirosaki University Graduate School of Medicine, 5 Zaifu-cho, Aomori, Hirosaki-shi, 036 8562, Japan

## Abstract

*Aim*. The goal of the study was to investigate quality of life (QOL) in adult patients with minimal change nephrotic syndrome (MCNS) and to test the relationship of QOL with the level of self-care. *Materials and Methods*. We distributed two questionnaires to 30 outpatients with MCNS. The MOS 36-item Short Form Health Survey (SF-36v2) was used to examine health-related QOL in comparison with normative data from the general Japanese population and a population with two chronic diseases. SF-36v2 consists of 36 questions classified into 8 subscales. We also used the Self-Care Behavior Scale for patients with chronic kidney disease (CKD), which consists of 31 questions with 4 subscales. *Results*. The SF-36v2 social functioning subscale was most impaired and bodily pain was least affected in patients with MCNS. The self-care subscales of information/communication and positive behavior had positive correlations with the QOL subscales of mental health (*P<0.05*) and vitality (*P<0.05*). The correlation between social functioning and information/communication was close to significant (*P=0.051*). *Conclusion*. In MCNS, social functioning was particularly impaired. Our results suggest that better self-care can have a positive impact on QOL in patients with MCNS.

## 1. Introduction

Longer life expectancy and advances in medical care have increased the problems associated with chronic diseases in many countries, including Japan. Chronic diseases have a long duration and generally slow progression, and people with these diseases desire to live longer and to live better. For these reasons, quality of life (QOL) is an increasingly important issue in healthcare for chronic diseases.

We have an interest in studying QOL of patients with renal diseases, as a typical example of chronic disease. The daily life of patients with renal disease is often limited by factors that are common to other chronic diseases, and these factors can easily decrease QOL. Furthermore, the number of patients with renal diseases is increasing worldwide. In 2010, about 297,000 people were receiving hemodialysis in Japan, and globally more than 500 million people have some degree of chronic kidney disease (CKD) [1].

Previous studies of QOL of patients with renal diseases have mainly focused on dialysis patients. There has been one study of QOL of pediatric patients with nephrotic syndrome and QOL of their parents [2]; however, to our knowledge, there have been no studies of QOL in adult patients with nephrotic syndrome. The onset of minimal change nephrotic syndrome (MCNS) is usually sudden and many patients experience repeated relapses and have to take regular medications that cause various side effects. Also, since kidney function is usually not impaired, medical staff may underestimate the impact of MCNS on the QOL of the patient.

To examine QOL in patients with MCNS, we chose to use the Short Form-36 (SF-36). This is the most widely used health-related QOL scale, and many previous studies have examined QOL of patients with renal diseases using this scale. Several studies have also shown a relationship between self-care and QOL in hemodialysis patients, and other studies have examined the relationship between self-efficacy and QOL [3–7]. Therefore, the aim of our study was to investigate QOL and the extent of self-care in adult patients with MCNS, with the goals of clarifying the relationship between QOL and self-care and using the results to suggest how to improve QOL in this patient population.

## 2. Materials and Methods

Questionnaires were distributed to 30 outpatients with MCNS at the Hirosaki University Hospital. The patients (average age 45.5 ± 17 years old) included 16 males (50.2 ± 19 years old) and 14 females (40 ± 15 years old). The study was approved by the Committee for Medical Ethics of Hirosaki University. All patients participated voluntarily on assurance of anonymity, and none refused to complete the questionnaires.

The SF-36v2 (MOS 36-item Short Form Health Survey) and the Self-Care Behavior Scale in CKD were used as questionnaires, with addition of questions on the background of the patients. The SF-36v2 consists of 8 subscales: physical functioning, physical role, bodily pain, general health, vitality, social functioning, emotional role, and mental health. All subscales are scored on a scale of 0 to 100 points, with higher values indicating better health-related QOL. The Self-Care Behavior Scale in CKD (Table 1) was developed by Ishikawa and Obata [8] and consists of 31 questions classified into 4 subscales: information/communication (7 questions), positive behavior (6 questions), diet/physical condition management (12 questions), and safety behavior (6 questions). Subjects chose the answer from 5 choices for each question: “I always do it,” “I often do it,” “I sometimes do it,” “I usually do not do so,” and “I do not do it at all”. Points from 1 to 5 were given for each answer, and the total points for each subscale and overall were calculated. Microsoft Office Excel 2007 and IBM SPSS Statistics 19 were used for statistical analysis. Data were analyzed using a Student's *t*-test or Pearson product-moment correlation coefficient test. A value of *P* < 0.05 or *P* < 0.001 was considered to be statistically significant.

## 3. Results

Of the 30 patients with MCNS, 27 lived with their family and 2 lived alone; 19 were employed and 9 were homemakers; 22 were taking medication and 7 were not; and 22 had experienced recurrence of MCNS and 5 had not had recurrence.

QOL scores for patients with MCNS compared with Japanese normative data from 2007 for the general population and for patients two or more chronic diseases are shown in Table 2. The SF-36 health-related QOL score was obtained from scores for all 8 subscales (physical functioning, physical role, bodily pain, general health, vitality, social functioning, emotional role and mental health), based on a 0- to 100-point scale with higher values indicating better QOL. The physical and mental component scores are calculated as summaries of multiple SF-36 subscales. For comparison, in the normative data for the general Japanese population, each subscale was given an average score of 50 and a standard deviation of 10. In the patients with MCNS, the score for bodily pain (*P* < 0.05) was significantly higher, and those for general health (*P* < 0.05) and social functioning (*P* < 0.001) were significantly lower than the Japanese averages. The scores for physical functioning (*P* < 0.001) and bodily pain (*P* < 0.001) and the physical component summary score (*P* < 0.05) were significantly higher in patients with MCNS compared to the averages for patients with two or more chronic diseases, but the score for social functioning (*P* < 0.001) was significantly lower in patients with MCNS.

**Figure 1:**
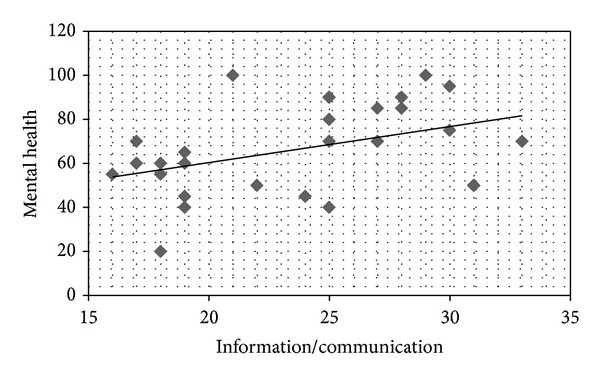
Correlation between mental health and information/communication. *r* = 0.413, *P* = 0.026.

**Figure 2:**
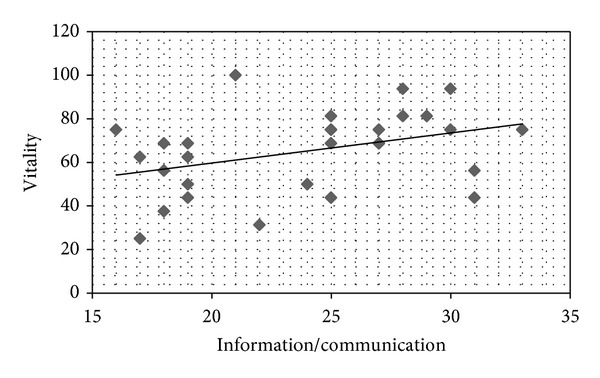
Correlation between vitality and information/communication. *r* = 0.374, *P* = 0.046.

**Figure 3:**
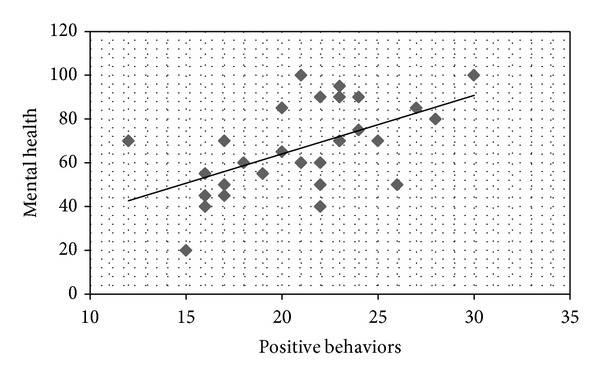
Correlation between mental health and positive behaviors. *r* = 0.559, *P* = 0.002.

**Figure 4:**
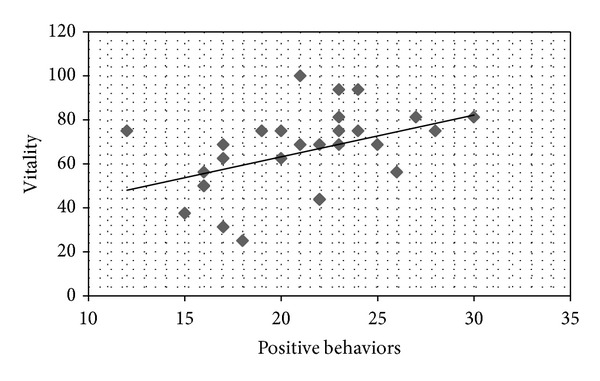
Correlation between vitality and positive behaviors. *r* = 0.429, *P* = 0.020.

On the Self-Care Behavior Scale in CKD, a higher score indicates greater self-care. Correlation coefficients were calculated between each SF-36 QOL subscale and the Self-Care Behavior scores. Positive correlations were found between mental health and information/communication (*r* = 0.413,  *P* < 0.05; Figure 1); vitality and information/communication (*r* = 0.374, *P* < 0.05; Figure 2); mental health and positive behavior (*r* = 0.559, *P* < 0.05; Figure 3); and vitality and positive behavior (*r* = 0.429, *P* < 0.05; Figure 4). Social functioning and information/communication showed a trend for a correlation, but this did not reach significance (*r* = 0.366, *P* = 0.051). There were no other correlations between SF-36 QOL subscales and Self-Care Behavior scores.

## 4. Discussion

The results of the study revealed unique changes in QOL in patients with MCNS and a relationship between the levels of QOL and self-care behavior. The patients had lower QOL subscale scores for general health and social functioning compared with the general Japanese population, with a particularly low score for social functioning that was even lower than that in Japanese normative data for patients with two or more chronic diseases. In contrast, the score for bodily pain was higher than that for patients with two or more chronic diseases and for the general Japanese population, showing that MCNS is a disease without pain. QOL for other attributes did not differ significantly for patients with MCNS compared with the general population. Among the self-care subscales, scores for information/communication and positive behavior had positive correlations with the QOL subscales of mental health and vitality.

In the United States, it has been shown that hemodialysis patients have lower QOL scores than CKD patients and that CKD patients have lower QOL scores than the general population for all items except mental health [9]. Rüth et al. found that pediatric patients with nephrotic syndrome had lower scores for social functioning compared with healthy children using another QOL scale [2]. The lower scores for social functioning were significantly related to steroid dependency and cyclophosphamide therapy among illness-related variables and were also related to the number of relapses, although without a significant association [2].

Tsay and Healstead found a negative correlation of QOL scores with depression and a positive correlation with self-efficacy in CKD patients [3]. Self-care education has been shown to improve laboratory data in several chronic diseases [10, 11]. However, one study in Japanese patients with renal diseases showed that scores for dietary self-assessment did not match with actual data for the serum urea nitrogen/urea creatinine ratio.

Our results suggest that nephrotic syndrome has an impact on social functioning of patients with MCNS, and previous findings suggest that QOL may also be influenced by steroid dependency, method of treatment, and depression. However, MCNS has a benign prognosis, and QOL of patients with MCNS may be better than that of CKD patients. We suggest that healthcare professionals should consider improving self-care not only to improve chronic diseases but also to improve self-efficacy for better QOL in these patients. In conclusion, our findings suggest that patients with MCNS have lower QOL based on low social functioning and that QOL is related to the positive behavior and thoughts of the patients. These results also show that healthcare professionals should be conscious of the QOL of patients with MCNS.

## Figures and Tables

**Table 1:** A scale to assess the practice of self-care behaviors for chronic kidney disease [8].

Self-Care Behavior Scale in CKD patients	
(1) Information/communication	
(i) I will get understanding to my feelings by talking with friends, family, doctors and nurses.	
(ii) I will get advice from friends, family, doctors, and nurses in a pinch.	
(iii) I will seek doctors and family's explanation of question about my condition and symptoms.	
(iv) I will soothe feelings by talking to someone about fear of the disease.	
(v) I will go to the hospital with little change in physical condition.	
(vi) I know the risk of neglect or refusal to take medication.	
(vii) I try to understand the disease.	
(2) Positive behaviors	
(i) I think optimistically.	
(ii) I do what I want without force.	
(iii) I will participate in activities involving movement as possible.	
(iv) I work off my frustration appropriately.	
(v) I enjoy my leisure time as possible.	
(vi) I endeavor to exercise regularly.	
(3) Physical condition management	
(i) I will cook diet considered.	
(ii) I note the water intake to prevent dehydration.	
(iii) I eat the balanced diet.	
(iv) I have devised so fun to eat by using my favorite dishes and so forth.	
(v) I am careful to keep within bounds in daily lives.	
(vi) I will know how much exercise in a day.	
(vii) I endeavor to understand the physical condition every day.	
(viii) I live a regular life.	
(ix) I note the changes in the urine.	
(x) I am eating a meal to suit me.	
(xi) I measure my blood pressure.	
(xii) I keep the dosage of the medicine.	
(4) Safety behaviors	
(i) I decided to follow the treatment without any change in symptoms.	
(ii) I will break moderately even busy.	
(iii) I avoid sleep deprivation.	
(iv) I will stop the medication at my discretion.	
(v) I avoid excessive exercise.	
(vi) I rest on the day of exercise.	

**Table 2:** QOL scores compared with Japanese normative data (2007).

		NS patients data		Normative data			Normative data		
Measure	*n*			(general people)			(with more than two chronic diseases)		
		Mean (average)	SD	Mean	SD	*P* value	Mean (average)	SD	*P* value
Physical functioning	30	51.83	6.59	50	10	0.140	46.2	13.5	<0.001**
Physical role	30	47.86	11.06	50	10	0.297	47.2	12.0	0.747
Bodily pain	30	54.52	9.03	50	10	0.010*	46.3	10.5	<0.001**
General health	30	45.82	6.59	50	10	0.002*	46.1	10.1	0.818
Vaitality	30	51.65	9.82	50	10	0.365	48.5	10.5	0.089
Social functioning	30	39.15	8.64	50	10	<0.001**	48.1	11.3	<0.001**
Emotional role	30	49.27	10.91	50	10	0.717	47.9	11.6	0.496
Mental health	30	47.89	11.00	50	10	0.301	48.8	10.7	0.652
Physical component summary	30	49.60	9.23	50	10	0.815	45.9	10.6	0.036*
Mental component summary	30	47.51	9.70	50	10	0.70	49.3	11.0	0.319

*P < 0.05, **P < 0.001.
